# Association of Treatments With Prevalence and Incidence of Periodontitis

**DOI:** 10.1002/cre2.70452

**Published:** 2026-09-08

**Authors:** Anna Liisa Suominen, Tuomas Saxlin, Marja‐Leena Lamidi, Virva Vänttinen, Johanna Pasanen, Tiina Laatikainen

**Affiliations:** ^1^ Institute of Dentistry University of Eastern Finland Kuopio Finland; ^2^ Oral and Maxillofacial Teaching Unit, Wellbeing Services County of Northern Savo Kuopio Finland; ^3^ Wellbeing Services County of North Karelia—Siun sote Joensuu Finland; ^4^ Institute of Public Health and Clinical Nutrition University of Eastern Finland Kuopio Finland

**Keywords:** ICD‐10 diagnosis, incidence, periodontitis, prevalence, register, therapeutics

## Abstract

**Objectives:**

The aim was to evaluate how treatment supplied in public oral health care services is associated with the prevalence and incidence of periodontitis.

**Methods:**

The data for this study comprised patients aged 20 years or older who had visited publicly funded oral health care services in the Wellbeing Services County of North Karelia (Siun sote) during the years 2021–2022 (*N* = 50,469). These patients were retrospectively followed in the patient register starting from 2014. Associations of treatments received during the follow‐up period (years 2014–2020) with ICD‐10 diagnoses of chronic and acute periodontitis at follow‐up (in 2021–2022) were analyzed by logistic regressions.

**Results:**

The prevalence of diagnoses of both chronic and acute periodontitis in 2021–2022 was quite low. The number of years of having received different treatments in 2014–2020 was associated with the odds of being diagnosed with periodontitis in 2021–2022. In the case of chronic periodontitis, an increasing number of years with preventive and restorative treatments was particularly associated with lower prevalence and incidence. Conversely, an increasing number of years with periodontal treatments and tooth extractions, in addition to earlier diagnosis, were associated with higher prevalence and incidence. For acute periodontitis, in contrast, an increasing number of years with preventive care among all participants and periodontal treatment in incident cases appeared to slightly increase the odds, whereas more years of examinations and tooth extractions were associated with reduced odds. In addition, smoking and diagnosis of diabetes mellitus were associated with higher odds of periodontitis diagnosis.

**Conclusions:**

While the association of treatments was similar for prevalence and incidence, it differed between chronic and acute periodontitis, highlighting their distinct pathophysiological profiles. Although preventive care is offered less frequently than other treatments and often for reasons unrelated to periodontal health, it shows a positive impact in reducing the risk of chronic but not acute periodontitis.

## 1 Introduction

Periodontitis, a chronic inflammatory condition affecting the tissues surrounding the teeth, is the most prevalent oral condition globally after untreated dental caries in permanent teeth (Bernabe et al. 2025). Also in Finland, periodontal diseases, including gingivitis and periodontitis, are common among adults (Suominen et al. 2025). The professional examination and treatment of oral diseases are expensive both to individuals and society due to direct costs and due to productivity losses (Jevdjevic and Listl 2025). Periodontal care traditionally includes regular examination, non‐surgical (e.g., supragingival scaling and polishing, and subgingival scaling) and surgical periodontal treatments. The purpose is to integrate diagnostic (examinations, check‐ups), preventive (e.g., oral hygiene instructions), and therapeutic (e.g., professional periodontal instrumentation) measures (Herrera et al. 2022; Sanz et al. 2020).

In addition to being cost‐effective, the assumption is that the greatest long‐term health benefits are derived from preventive actions, lifestyle changes, and preventive services (Working group set up by the Finnish Medical Society Duodecim and the Finnish Dental Society 2025). Regular dental check‐ups, examination of periodontal health, and personalized counseling are considered essential for preventing periodontitis and identifying it in its early stages, before significant tissue damage occurs and treatment costs escalate. However, evidence supporting this is scarce but would be needed for decision‐making and prioritization of the limited available resources because of the high prevalence of the condition (Jevdjevic and Listl 2025). Nevertheless, frequent annual or even biannual dental visits have become common practice in many affluent Western societies, despite the lack of strong supporting evidence (Lamont et al. 2018; Fee et al. 2020)

So far, patient registers, that is, real‐world data, are underutilized even though such information would have clearly added value in assessing the impact of oral health care services in developing the processes in disease management and in allocating resources. In the US (Taylor et al. 2022), it was found that preventive dental visits were associated with fewer subsequent non‐preventive visits and lowered dental expenditures in low‐income adult populations. Although patients value routine scaling and polishing and are willing to pay for them, these services alone do not provide substantial clinical benefits for adults with regular dental care and without severe periodontitis (Clarkson et al. 2021; Ramsay et al. 2018). Routine scaling and polishing were also deemed to offer only limited clinical benefits for adults with no or early periodontal disease in a review by Matthews and Al‐Waeli (Matthews and Al‐Waeli 2024). However, for individuals with periodontitis, they may help to reduce some health care expenses and tooth loss, the ultimate consequence of the disease.

Register‐based studies on periodontal treatment effectiveness have generally found that receiving periodontal treatment is associated with better clinical periodontal status (Haukka et al. 2025; Kocher et al. 2025), lower tooth loss (Chan et al. 2016; Raedel et al. 2019; Kocher et al. 2022; Raittio et al. 2025; Raittio and Baelum 2025), and less health care expenses (Kao et al. 2021; Smits et al. 2020). However, little is known about the association between current, expensive oral health care services and the prevalence and incidence of periodontal diseases. To address this evidence gap, the present study aimed to investigate how treatment provided in public oral health care services is associated with the prevalence and incidence of chronic and acute periodontitis.

## 2 Methods

### 2.1 Study Design

The present study was conducted as a retrospective, longitudinal study based on data from the patient information system of the Wellbeing Services County of North Karelia (Siun sote) in Finland. Siun sote has operated since 2017, covering 13 municipalities and 163,537 inhabitants at the end of 2020. Since 2011, Siun sote has used a unified patient information system (Mediatri) in primary health care, specialized medical care, and social services. The system is structured, allowing for the comprehensive collection of diverse data on a register basis. Visit and treatment data have been comprehensively and reliably available retrospectively since 2014.

**Figure 1:**
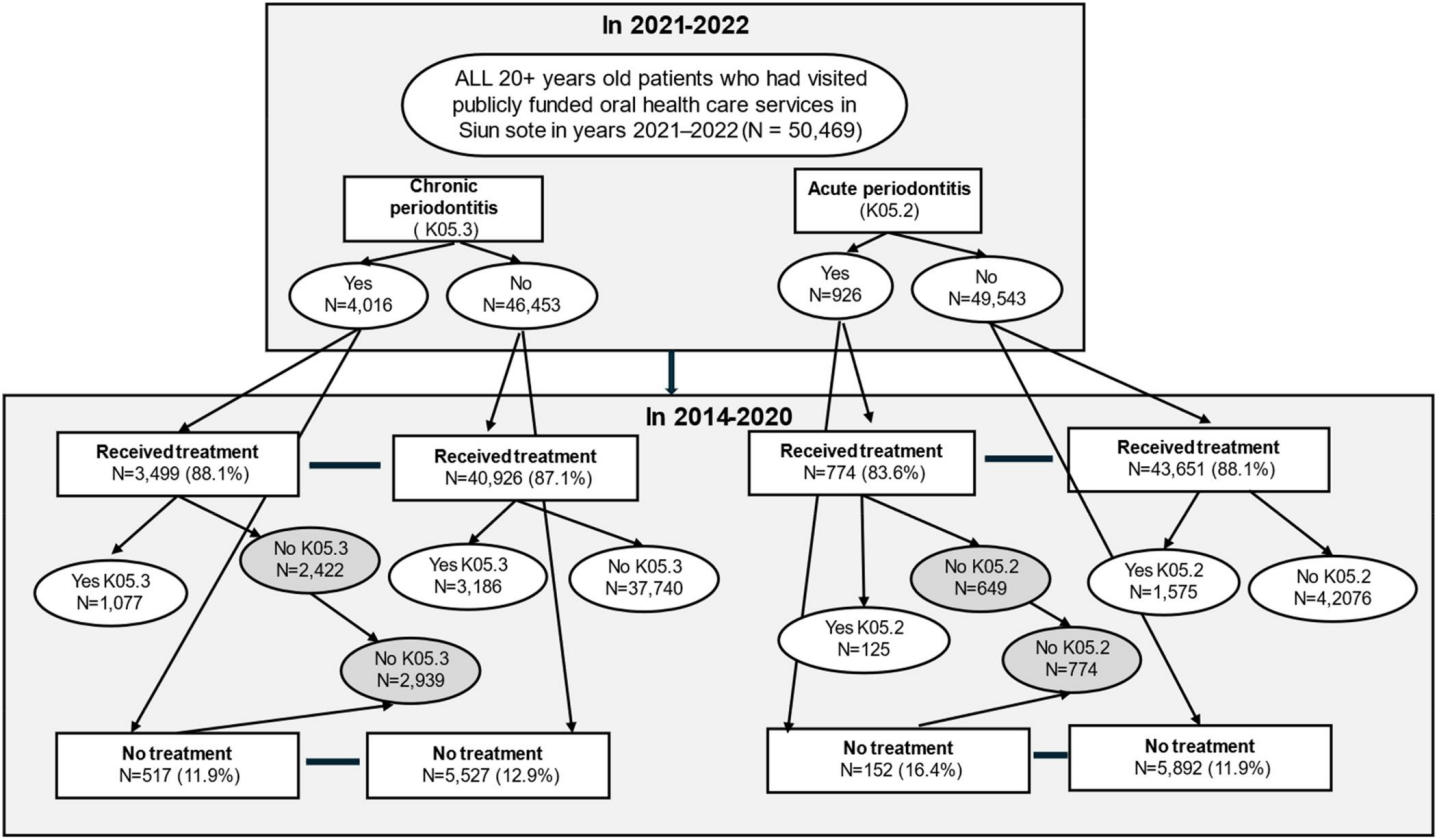
Flow chart describing the design and participants of the study. Incident cases in 2021–2022 indicated by gray background.

The data of this study included patients aged 20 years or older who had visited publicly funded oral health care services in Siun sote, that is, had an appointment with a dentist, dental hygienist, or dental nurse, in the years 2021–2022 (*N* = 50,469). These patients were retrospectively followed through the register, and data from each of their visits between 2014 and 2020 were retrieved (Figure 1).

### 2.2 Outcomes

These patients were classified based on the ICD‐10 classification of “Diseases of the oral cavity, salivary glands, and jaws” (Classification of Diseases 2024). The outcomes of this study were defined as having had a chronic periodontitis (ICD‐10 code K05.3) or acute periodontitis (K05.2) diagnosis in 2021–2022, including both the primary and secondary diagnoses. The use of diagnostic codes of periodontal diseases in Finland is guided nationally by the Current Care Guidelines for Periodontitis (Working group set up by the Finnish Medical Society Duodecim and the Finnish Dental Society 2025), which are aligned with the disease classification proposed by the 2017 World Workshop on the Classification of Periodontal and Peri‐Implant Diseases and Conditions (R Core Team 2021) and with the recommendations of the European Federation of Periodontology S3 level clinical practice guideline (Sanz et al. 2020).

### 2.3 Exposures

Oral health care treatment received in 2014–2020 was used as the exposure. Treatment measures in public oral health care services in Finland are recorded according to the classification maintained by the Finnish Institute for Health and Welfare (THL). The oral health care procedure classification consists of codes specific to the field of oral health care. These codes are used when preparing patient records and serve to describe the content of the care provided. The classification is used in both the public and private sectors. The following main categories were used: SA—Examinations, SC—Preventive measures including health counseling (e.g., smoking and alcohol), nutritional analysis, self‐care education, necessary fluoride or other similar treatment, removal of plaque and plaque retention areas, as well as an assessment of preventive care, SD—Treatment of periodontal diseases including management of inflammation, tissue changes, and occlusion, periodontal splinting, as well as patient guidance and maintenance therapy related to disease management (also divided into treatment of gingival alterations and/or uncomplicated periodontitis [SDA01–SDA05] vs. complicated periodontitis [SDA12–SDA14]), SF—Restorative treatments, and EBA—Tooth extractions. In the analysis, treatments were considered both as a binary variable (received at least once vs. not received) and as the number of years during the follow‐up period in which they were received.

### 2.4 Covariates

Covariate information was gathered from the register and included age, sex, smoking status at follow‐up (categorized as non‐smokers, former, or current smokers), earlier diagnoses (ICD‐10) of periodontal diseases (K05.0–K05.3), dental caries (K02.1), and other diseases during the follow‐up period as follows: type 1 and 2 diabetes mellitus (DM) (E10–E11), schizophrenia, schizotypal, delusional, and other non‐mood psychotic disorders (F20–F29 excluding F23), mood (affective) disorders (F30–F39), ischemic heart diseases (I20–I25), cerebrovascular diseases (I60–I69), diseases of esophagus, stomach, and duodenum (K20–K31), Crohn's disease and ulcerative colitis (K50–K51), inflammatory polyarthropathies (M05–M14), and systemic connective tissue disorders (M30–M36).

### 2.5 Statistical Analysis

**Figure 2:**
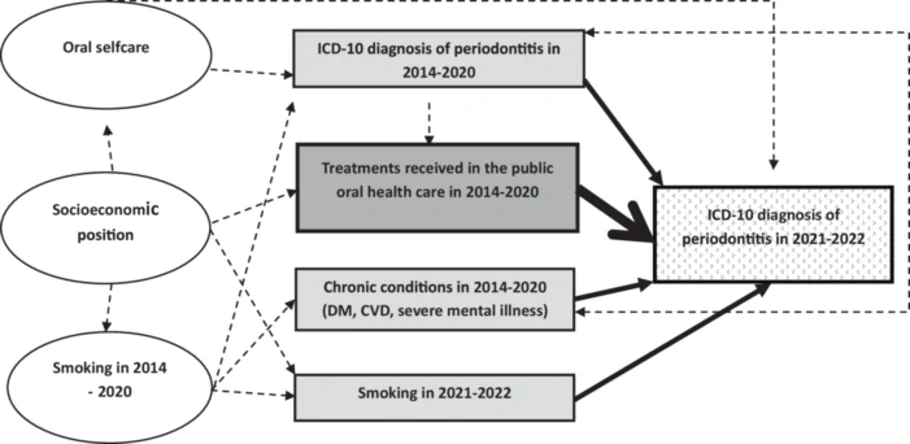
A Directed Acyclic Graph (DAG) that was used as the basis for studying the association between treatments received in 2014–2020 (exposure) and ICD‐10 diagnoses of periodontitis in 2021–2022 (outcome). A solid thick line represents the path between the exposure and the outcome, while solid thin lines indicate paths from variables used for adjustment. Dashed lines denote factors and paths that were not available in these data.

Binary logistic regression analyses were first used to study the associations of the exposure and the covariates separately with the occurrence of chronic or acute periodontitis diagnosis in 2021–2022, adjusted for sex, age group, and their interactions. These associations were based on a Directed Acyclic Graph (DAG) shown in Figure 2. Separate models were fitted for (a) all patients in the data in 2021–2022, (b) for all patients in 2021–2022 and with treatments received in Siun sote oral health care in 2014–2020, (c) for the incident cases in 2021–2022, defined as individuals without a previous diagnosis of chronic or acute periodontitis during the period 2014–2020, and (d) for the incident cases in 2021–2022, defined as those without earlier diagnosis of chronic or acute periodontitis in 2014–2020 and had received treatments in Siun sote oral health care in 2014–2020. Secondly, multivariate models were implemented to investigate the association of the exposure and outcomes, with smoking, earlier chronic and acute periodontitis diagnoses, and ICD 10‐diagnoses for type 1 or 2 DM, ischemic heart diseases, and mood (affective) disorders as covariates, and, in addition, adjusted for sex, age group, and their interactions. Separate models were fitted for all patients in the data in 2021–2022 and for the incident cases in 2021–2022 who had received treatments in Siun sote oral health care in 2014–2020. In addition, stratified analyses by age group and sex for chronic and acute periodontitis diagnoses among all participants were implemented. R statistical software was used in the analyses (R Core Team 2021).

## 3 Results

### 3.1 Description of the Study Participants

About 31% of the inhabitants of Siun sote had visited oral health care in 2021–2022. Their mean age was 55 (SD 18, median 58, Q1 40, Q3 69, maximum 104), and 45.1% (*N* = 22,752) were men.

Of the patients, 8.0% had a diagnosis of chronic periodontitis and 1.8% of acute periodontitis in 2021–2022. In 2014–2020, both chronic and acute periodontitis patients who visited oral health care in 2021–2022, were more frequently examined or received restorative and periodontal treatments than other patients on average. Preventive measures, however, were provided to less than one‐third of these patients in 2014–2020 (Table 1).

**Table 1:** Characteristics of all visitors and those with chronic (ICD‐10 code K05.3) or acute periodontitis (K05.2) diagnoses in the public oral health care services in the Wellbeing Services County of North Karelia (Siun sote), Finland, in 2021–2022.

Variable	All	Chronic periodontitis diagnosis in 2021–2022	Acute periodontitis diagnosis in 2021–2022
*N* (%)	50,469 (100)	4,016 (8.0)	926 (1.8)
Sex, % of men	45.1	48.9	41.6
Age, median	58	62	34
Smoking status in 2021–2022, **%**			
Never smokers	77.1	66.7	66.5
Former smokers	12.4	16.4	15.9
Current smokers	10.5	16.9	17.6
Gingivitis and periodontitis diagnoses (ICD‐10) in 2014–2020, %			
Chronic periodontitis	8.4	26.8	12.9
Acute periodontitis	3.4	6.3	13.5
Chronic gingivitis	4.5	4.4	4.1
Acute gingivitis	1.4	1.4	1.4
Treatments received in 2014–2020, % / mean *n* of years (max *n* of years 7, if missing)			
Any	88.0/3.34	87.2/3.39	83.6/2.82
Examinations (SA)	82.4/2.21	83.4/2.32	78.3/1.94
Preventive measures (SC)	25.3/0.37	24.0/0.34	28.7/0.44
Treatment of periodontal diseases (SD)	59.5/1.26	65.9/1.61	52.8/1.03
Treatment of uncomplicated periodontitis and/or gingival alterations (SDA01–SDA05)	53.3/0.94	60.0/1.19	47.8/0.79
Treatment of complicated periodontitis and/or gingival alterations (SDA12–SDA14)	26.0/0.35 (6)	32.8/0.50 (6)	20.4/0.27 (3)
Restorative treatments (SF)	74.6/2.28	71.3/2.13	62.2/1.55
Tooth extractions (EBA)	39.3/0.60	49.6/0.83	36.6/0.52 (5)
Other diagnoses (ICD‐10) in 2014–2020, %			
Type 1 or 2 diabetes mellitus (E10–E11)	10.9	14.9	5.8
Ischemic heart diseases (I20–I25)	5.9	7.9	3.1
Cerebrovascular diseases (I60–I69)	2.3	2.7	1.1
Inflammatory polyarthropathies (M05–M14)	3.3	4.1	1.5
Systemic connective tissue disorders (M30–M36)	1.0	1.1	0.9
Schizophrenia, schizotypal, delusional, and other non‐mood psychotic disorders (F20–29 excluding F23)	3.8	5.5	3.5
Mood [affective] disorders (F30–F39)	3.8	4.3	3.3
Stomach and duodenum (K20–K31)	2.1	2.0	1.5
Crohn's disease and ulcerative colitis (K50–K51)	1.3	1.1	0.8

The numbers of incident cases in 2021‐2022 (Figure 1), that is, those without corresponding earlier diagnosis in 2014–2020, were 2939 (6.4%) for chronic periodontitis and 801 (1.6%) for acute periodontitis. Among the age groups (20–34, 35–49, 50–64, 65–79, 80–), chronic periodontitis diagnosis was the most common among the 65–79‐year‐old men (11.1%), and the most uncommon among the 35–49‐year‐old women (4.7%). Acute periodontitis diagnosis, instead, was the most common among the youngest (20–34‐year‐old) women (6.4%) at follow‐up (Table 2).

**Table 2:** Prevalences (%) of chronic (ICD‐10 code K05.3) and acute periodontitis (K05.2) diagnoses in 2021–2022 by sex and age groups among the visitors in the public oral health care services in the Wellbeing Services County of North Karelia (Siun sote), Finland.

	Age group					
	20–34	35–49	50–64	65–79	80–	Total
	%					
Chronic periodontitis diagnosis in 2021–2022						
ALL (n)	(589)	(594)	(1,102)	(1,405)	(326)	(4016)
Total	7.0	5.6	8.0	10.2	8.2	8.0
Men	6.8	6.8	8.6	11.1	9.4	8.6
Women	7.1	4.7	7.6	9.4	7.4	7.4
Without earlier chronic periodontitis diagnosis in 2014–2020						
Total	6.6	4.9	6.0	7.8	6.3	6.4
Men	6.3	5.9	6.5	8.8	7.2	7.0
Women	6.9	4.1	5.5	7.0	5.7	5.8
Acute periodontitis diagnosis in 2021–2022						
ALL (*n*)	(467)	(160)	(131)	(137)	(31)	(926)
Total	5.5	1.5	1.0	1.0	0.8	1.8
Men	4.6	1.5	0.9	1.1	0.6	1.7
Women	6.4	1.6	1.0	0.9	0.9	2.0
Without earlier acute periodontitis diagnosis in 2014–2020						
Total	5.1	1.3	0.9	0.9	0.7	1.6
Men	4.3	1.3	0.9	1.1	0.7	1.5
Women	5.8	1.4	1.0	0.8	0.8	1.7

### 3.2 Age, Sex, and Their Interaction Adjusted Associations of Treatments With Periodontitis

Visits to oral health care, either at all or over a greater number of years, in 2014–2020, were associated with lower odds of having both chronic (OR 0.90 [0.82–0.99]) or acute (OR 0.76 [0.64–0.91]) periodontitis diagnosis in 2021–2022 (Tables 3 and 4). This was seen both in all patients and in the incident cases. Restorative treatments were associated with lower odds of chronic periodontitis diagnoses among all participants (OR 0.79 [0.79–0.85]) and in incident cases (OR 0.71 [0.65–0.77]), while dental examinations (OR 0.81 [0.74–0.89]) and preventive measures (OR 0.80 [0.73–0.88]) were additionally associated with lower odds in the incident‐case analyses. Conversely, periodontal treatments (OR 1.33 [1.25–1.43]) and tooth extractions (OR 1.27 [1.23–1.31]) exhibited higher odds of having chronic periodontitis in 2021–2022, with similar associations in incident cases. For acute periodontitis, examinations (OR 0.78 [0.67–0.92]), restorative treatments (OR 0.72 [0.63–0.82]), and a higher number of years of tooth extractions (OR 0.91 [0.84–0.99]) were associated with the acute diagnoses, with similar associations in incident cases.

**Table 3:** Sex, age group, and their interaction adjusted odds ratios (OR) with 95% confidence intervals (95% CI) for occurrence of chronic periodontitis diagnosis (ICD‐10 code K05.3) in 2021–2022 by logistic regression, separate models for each variable.

	All participants	Participants having received treatments in Siun sote oral health care in 2014–2020	Incident cases, individuals without a chronic periodontitis diagnosis in 2014–2020	Incident cases, individuals without a chronic periodontitis diagnosis in 2014–2020 and had received treatments in Siun sote oral health care in 2014–2020
No. of patients with chronic periodontitis diagnosis (ICD‐10) in 2021–2022/Total (%)	4,016/50,469 (8.0)	3,499/44,425 (7.9)	2,939/46,206 (6.4)	2,422/40,162 (6.0)
	**OR (95% CI)**			
Variable				
In 2021‐2022				
Smoking (ref. never smokers)				
Current	**2.25 (2.05–2.47)**	**2.41 (2.19–2.65)**	**2.03 (1.82–2.27)**	**2.21 (1.96–2.49)**
Former	**1.75 (1.60–1.92)**	**1.86 (1.69–2.05)**	**1.62 (1.46–1.82)**	**1.75 (1.56–1.96)**
In 2014‐2020				
Gingivitis and periodontitis diagnoses (ICD‐10)				
Chronic periodontitis	**4.81 (4.45–5.21)**	**5.09 (4.70–5.52)**	**—**	**—**
Acute periodontitis	**2.35 (2.04–2.71)**	**2.42 (2.10–2.79)**	**1.98 (1.65–2.39)**	**2.10 (1.74–2.53)**
Chronic gingivitis	0.99 (0.85–1.16)	1.01 (0.86–1.18)		
Acute gingivitis	1.11 (0.84–1.46)	1.12 (0.84–1.48)		
Treatments received				
Any treatment, yes/no	**0.90 (0.82–0.99)**		**0.69 (0.62–0.76)**	
Years of treatment	1.00 (0.99–1.02)	1.02 (1.00–1.04)	**0.94 (0.93–0.96)**	**0.97 (0.95–0.99)**
Examinations (SA), y/n	1.08 (0.99–1.17)	**1.59 (1.35–1.89)**	**0.81 (0.74–0.89)**	**1.22 (1.02–1.45)**
Years of examinations (SA)	**1.04 (1.02–1.06)**	**1.06 (1.04–1.08)**	**0.96 (0.94–0.98)**	1.00 (0.97–1.03)
Preventive measures (SC), y/n	0.94 (0.87–1.02)	0.95 (0.88–1.03)	**0.80 (0.73–0.88)**	**0.85 (0.77–0.93)**
Years of preventive measures (SC)	**0.95 (0.91–1.00)**	0.96 (0.91–1.00)	**0.90 (0.85–0.95**	**0.92 (0.87–0.98)**
Periodontal treatment (SD), y/n	**1.33 (1.25–1.43)**	**1.52 (1.41–1.65)**	1.03 (0.95–1.11)	**1.24 (1.13–1.36)**
Years of periodontal treatments (SD)	**1.15 (1.13–1.18)**	**1.18 (1.16–1.20)**	**1.04 (1.01–1.07)**	**1.08 (1.06–1.11)**
Restorative treatments (SF), y/n	**0.79 (0.74–0.85)**	**0.74 (0.68–0.82)**	**0.71 (0.65–0.77)**	**0.78 (0.70–0.86)**
Years of restorative treatments (SF)	**0.94 (0.93–0.96)**	**0.94 (0.92–0.96)**	**0.92 (0.90–0.94)**	**0.94 (0.92–0.96)**
Tooth extractions (EBA), y/n	**1.54 (1.44–1.64)**	**1.67 (1.56–1.79)**	**1.16 (1.08–1.25)**	**1.31 (1.21–1.42)**
Years of tooth extractions (EBA)	**1.27 (1.23–1.31)**	**1.31 (1.26–1.35)**	**1.11 (1.06–1.16)**	**1.17 (1.12–1.22)**
Other diagnoses (ICD‐10)				
Type 1 or 2 diabetes mellitus (E10–E11)	**1.29 (1.17–1.42)**	**1.30 (1.17–1.43)**	**1.20 (1.07–1.35)**	**1.21 (1.06–1.37)**
Ischemic heart diseases (I20–I25)	**1.17 (1.02–1.33)**	**1.16 (1.01–1.33)**	1.14 (0.98–1.34)	
Schizophrenia, schizotypal, delusional, and other non‐mood psychotic disorders (F20–F29 excluding F23)	**1.53 (1.33–1.77)**	**1.63 (1.40–1.90)**	**1.44 (1.21–1.72)**	**1.56 (1.29–1.87)**
Mood (affective) disorders (F30–F39)	**1.22 (1.04–1.44)**	**1.27 (1.07–1.50)**	1.17 (0.97–1.42)	**1.24 (1.02–1.52)**

*Note:* Model 1: *p*‐value < 0.001 for sex and age group and 0.008 for their interaction; < 0.001 for home municipality. Statistically significant findings in bold.

**Table 4:** Sex, age group, and their interaction adjusted odds ratios (OR) with 95% confidence intervals (95% CI) for occurrence of acute periodontitis diagnosis (ICD‐10 code K05.2) in 2021–2022 by logistic regression, separate models for each variable.

	All participants	Participants having received treatments in Siun sote oral health care 2014–2020	Incident cases, individuals without acute periodontitis diagnosis in 2014–2020	Incident cases, individuals without acute periodontitis diagnosis in 2014‐2020 and who had received treatments in Siun sote oral health care in 2014–2020
No. of patients with acute periodontitis diagnosis (ICD‐10) in 2021–2022/Total (%)	926/50,469 (1.8)	774/44.425 (1.7)	801/48,769 (1.6)	649/42,725 (1.5)
	**OR (95% CI)**			
Variable				
In 2021–2021				
Smoking (ref. never smokers)				
Current	**1.34 (1.26‐1.42)**	**1.32 (1.24–1.40)**	**1.36 (1.27–1.45)**	**1.34 (1.24–1.44)**
Former	**1.26 (1.19–1.33)**	**1.25 (1.18–1.32)**	**1.28 (1.20–1.36)**	**1.28 (1.20–1.37)**
In 2014‐2020				
Periodontitis diagnoses (ICD‐10)				
Chronic periodontitis	**1.70 (1.40–2.08)**	**1.80 (1.47–2.20)**	**1.74 (1.39–2.18)**	**1.88 (1.50–2.36)**
Acute periodontitis	**2.70 (2.20–3.31)**	**2.91 (2.37–3.58)**	**—**	**—**
Treatments received				
Any treatment, yes/no	**0.76 (0.64–0.91)**		**0.68 (0.57–0.82)**	
Years of treatment	**0.93 (0.90–0.96)**	**0.94 (0.90–0.98)**	**0.92 (0.89–0.95)**	**0.93 (0.89–0.98)**
Examinations (SA), y/n	**0.78 (0.67–0.92)**	0.87 (0.65–1.17)	**0.70 (0.60–0.83)**	0.80 (0.59–1.08)
Years of examinations (SA)	**0.92 (0.88–0.96)**	**0.93 (0.89–0.98)**	**0.89 (0.85–0.93)**	**0.91 (0.86–0.96)**
Preventive measures (SC), y/n	1.01 (0.87–1.16)	1.07 (0.92–1.25)	0.99 (0.84–1.16)	1.09 (0.92–1.28)
Years of preventive measures (SC) years	1.01 (0.93–1.09)	1.03 (0.95–1.12)	0.99 (0.91–1.08)	1.03 (0.95–1.13)
Periodontal treatment (SD), y/n	0.90 (0.79–1.03)	0.97 (0.84–1.13)	0.87 (0.76–1.01)	1.00 (0.84–1.17)
Years of periodontal treatment (SD)	0.97 (0.92–1.02)	1.00 (0.94–1.05)	0.97 (0.92–1.02)	1.01 (0.95–1.07)
Restorative treatments (SF), y/n	**0.72 (0.63–0.82)**	**0.73 (0.62–0.86)**	**0.71 (0.61‐0.82)**	**0.77 (0.64–0.93)**
Years of restorative treatments (SF)	**0.88 (0.85–0.92)**	**0.88 (0.85–0.92)**	**0.89 (0.85‐0.92)**	**0.90 (0.86–0.94)**
Tooth extractions (EBA), y/n	0.92 (0.80–1.05)	0.97 (0.84–1.13)	0.90 (0.78–1.05)	0.96 (0.87–1.05)
Years of tooth extractions (EBA)	**0.91 (0.84–0.99)**	0.94 (0.86–1.02)	**0.92 (0.84–1.00)**	0.99 (0.84–1.16)
Other diagnoses (ICD‐10)				
Type 1 or 2 diabetes mellitus (E10–E11)	0.87 (0.65–1.16)	0.91 (0.67–1.23)	0.87 (0.64–1.17)	0.91 (0.66–1.26)
Ischemic heart diseases (I20–I25)	1.05 (0.70–1.57)	1.07 (0.70–1.64)	1.07 (0.70–1.62)	1.09 (0.70–1.70)
Schizophrenia, schizotypal, delusional, and other non‐mood psychotic disorders (F20–29 excluding F23)	1.05 (0.73–1.50)	1.10 (0.75–1.60)	1.00 (0.67–1.49)	1.06 (0.69–1.61)
Mood (affective) disorders (F30–F39)	0.85 (0.59–1.22)	0.90 (0.61–1.31)	**0.62 (0.39–0.98)**	0.65 (0.40–1.05)

*Note:* Model 1: *p*‐value 0.008 for sex, < 0.001 for age group and 0.060 for their interaction. Statistically significant findings in bold.

### 3.3 Associations of Treatments With Periodontitis in Multivariate Regression Analyses

For chronic periodontitis, a higher number of years of preventive (OR 0.92 [0.87–0.96]) or restorative treatments (OR 0.89 [0.91–0.93]) in 2014–2020 were associated with lower odds for the diagnosis in 2021–2022 (Table 5). Similar associations were observed for the incident cases. Conversely, individuals who had received periodontal treatment (OR 1.12 [1.10–1.15]) or undergone tooth extractions (OR 1.12 [1.08–1.16]) in 2014–2020 exhibited higher odds for the diagnosis in 2021–2022, similarly for incident cases.

**Table 5:** Adjusted^a^ odds ratios (OR) with 95% confidence intervals (95% CI) for the occurrence of chronic periodontitis (ICD‐10 code K05.3) or acute periodontitis (K05.2) diagnoses in 2021–2022 by multivariate logistic regressions.

	Chronic periodontitis		Acute periodontitis	
	All participants	Incident cases, individuals without a chronic periodontitis diagnosis in 2014–2020 and who had received treatments in Siun sote oral health care in 2014–2020	All participants	Incident cases, individuals without a acute periodontitis diagnosis in 2014–2020 and who had received treatments in Siun sote oral health care in 2014–2020
No. of patients with periodontitis diagnosis (ICD‐10) in 2021–2022/Total (%)	4,016/50,469 (8.0)	2,422/40,162 (6.0)	926/50,469 (1.8)	649/42,725 (1.5)
	**OR (95% CI)**			
In 2021–2022				
Smoking (ref. never smokers)				
Current	**1.85 (1.68–2.04)**	**2.05 (1.81–2.31)**	**1.46 (1.21–1.75)**	**1.61 (1.30–2.00)**
Former	**1.56 (1.42–1.72)**	**1.69 (1.50–1.90)**	1.17 (0.97–1.41)	1.20 (0.96–1.50)
In 2014‐2020				
Periodontitis diagnoses				
Chronic periodontitis	**3.79 (3.47–4.14)**	**—**	**1.72 (1.38–2.15)**	**1.98 (1.55–2.53)**
Acute periodontitis	**1.38 (1.18–1.61)**	**1.76 (1.45–2.12)**	**2.85 (2.28–3.55)**	**—**
Years of treatments				
Examinations (SA)	0.98 (0.95–1.01)	1.00 (0.96–1.04)	**0.94 (0.88–1.00)**	**0.92 (0.85–0.99)**
Preventive measures (SC)	**0.92 (0.87–0.96)**	**0.92 (0.87–0.97)**	**1.11 (1.02–1.21)**	**1.11 (1.01–1.22)**
Periodontal treatment (SD)	**1.12 (1.10–1.15)**	**1.12 (1.09–1.16)**	0.99 (0.93–1.05)	**1.01 (0.95–1.08)**
Restorative treatment (SF)	**0.89 (0.87–0.91)**	**0.91 (0.88–0.93)**	**0.91 (0.87–0.96)**	**0.93 (0.88–0.98)**
Tooth extractions (EBA)	**1.12 (1.08–1.16)**	**1.16 (1.10–1.21)**	**0.85 (0.77–0.94)**	0.95 (0.86–1.05)
Other diagnoses (ICD‐10)				
Type 1 or 2 diabetes mellitus (E10–E11)	**1.13 (1.02–1.25)**	1.09 (0.96–1.24)	0.87 (0.65–1.17)	0.88 (0.64–1.23)
Ischemic heart diseases (I20–I25)	1.06 (0.93–1.21)	1.08 (0.91–1.28)	1.08 (0.71–1.62)	1.10 (0.70–1.71)
Schizophrenia, schizotypal, delusional, and other non‐mood psychotic disorders (F20–F29 excluding F23)	1.17 (1.00–1.37)	1.18 (0.97–1.43)	1.02 (0.71–1.48)	1.03 (0.67–1.59)
Mood (affective) disorders (F30–F39)	1.00 (0.85–1.19)	1.05 (0.86–1.29)	0.80 (0.55–1.16)	0.62 (0.38–1.02)

*Note:* Statistically significant findings in bold.

^a^ Also for sex, age group, and their interaction.

For acute periodontitis, a higher number of years of examinations (OR 0.94 [0.88–1.0]), restorative treatments (OR 0.91 [0.87–0.96]), or tooth extractions (OR 0.85 [0.77–0.94]) in 2014–2020 were associated with lower odds for the diagnosis in 2021–2022 (Table 3). A higher number of years of preventive measures was an exception and was associated with higher odds for the diagnosis (OR 1.11 [1.02–1.21]). For the incident cases, associations were similar, but periodontal treatment was associated with higher odds for the diagnosis (OR 1.01 [0.95–1.08]).

Results from the age‐ and sex‐stratified analyses were consistent with the findings of the multivariable analyses (Tables 6 and 7). The only exception was the association between preventive treatments and a diagnosis of acute periodontitis. A higher number of years receiving preventive care was associated with increased odds of the diagnosis (OR 1.18, 95% CI 1.05–1.32) only among women under 65 years of age, whereas the association was not statistically significant in the other groups.

**Table 6:** Adjusted odds ratios (OR) with 95% confidence intervals (95% CI) for the occurrence of chronic (ICD‐10 code K05.3) periodontitis diagnoses in 2021–2022 among all participants by multivariate logistic regressions stratified by age group and sex.

	OR (95% CI)			
In 2021–2022	Men, aged < 65 years	Women, aged < 65 years	Men, aged 65+ years	Women, aged 65+ years
Smoking (ref. never smokers)				
Current	**1.65 (1.38–1.96)**	**2.32 (1.99–2.71)**	1.23 (0.93–1.63)	**1.93 (1.47–2.54)**
Former	**1.50 (1.28–1.76)**	**1.85 (1.57–2.18)**	1.25 (0.98–1.58)	**1.66 (1.29–2.14)**
In 2014–2020				
Periodontitis diagnoses				
Chronic periodontitis	**3.87 (3.26–4.60)**	**3.47 (2.92–4.12)**	**3.38 (2.79–4.09)**	**4.44 (3.70–5.33)**
Acute periodontitis	**1.53 (1.16–2.00)**	1.19 (0.93–1.52)	1.20 (0.73–1.98)	**1.97 (1.36–2.86)**
Years of treatments				
Examinations (SA)	1.00 (0.94–1.06)	0.96 (0.91–1.02)	1.01 (0.94–1.08)	0.98 (0.92–1.04)
Preventive measures (SC)	0.95 (0.86–1.05)	0.97 (0.89–1.05)	**0.84 (0.74–0.96)**	**0.86 (0.78–0.96)**
Periodontal treatment (SD)	**1.12 (1.06–1.18)**	**1.14 (1.09–1.19)**	**1.14 (1.08–1.20)**	**1.10 (1.05–1.15)**
Restorative treatment (SF)	**0.88 (0.84–0.92)**	**0.89 (0.85–0.93)**	**0.86 (0.82–0.90)**	**0.92 (0.88–0.97)**
Tooth extractions (EBA)	1.05 (0.98–1.13)	**1.08 (1.00–1.16)**	**1.19 (1.10–1.29)**	**1.19 (1.10–1.30)**
Other diagnoses (ICD‐10)				
Type 1 or 2 diabetes mellitus (E10–E11)	**1.30 (1.05–1.62)**	1.14 (0.89–1.46)	1.09 (0.92–1.30)	1.07 (0.89–1.30)
Ischemic heart diseases (I20–I25)	1.51 (1.09–2.09)	1.21 (0.69–2.11)	1.01 (0.83–1.22)	0.95 (0.73–1.23)
Schizophrenia, schizotypal, delusional, and other non‐mood psychotic disorders (F20–F29 excluding F23)	1.11 (0.85–1.44)	**1.79 (1.37–2.35)**	0.82 (0.54–1.24)	0.96 (0.65–1.41)
Mood (affective) disorders (F30–F39)	0.89 (0.63–1.25)	1.15 (0.90–1.47)	0.83 (0.46–1.51)	0.83 (0.55–1.25)

*Note:* Statistically significant findings in bold.

**Table 7:** Adjusted^1^ odds ratios (OR) with 95% confidence intervals (95% CI) for the occurrence of acute (ICD‐10 code K05.2) periodontitis diagnoses in 2021–2022 among all participants by multivariate logistic regressions stratified by age group and sex.

	OR (95% CI)			
In 2021–2022	Men, aged < 65 years	Women, aged < 65 years	Men, aged 65+ years	Women, aged 65+ years
Smoking (ref. never smokers)				
Current	1.34 (0.94–1.89)	**1.68 (1.32–2.14)**	0.76 (0.30–1.95)	1.22 (0.53–2.83)
Former	**1.47 (1.10–1.95)**	1.07 (0.81–1.41)	0.95 (0.45–2.01)	0.78 (0.31–1.98)
In 2014–2020				
Periodontitis diagnoses				
Chronic periodontitis	**1.61 (1.05–2.47)**	1.27 (0.89–1.81)	**4.08 (2.37–7.03)**	1.82 (0.99–3.33)
Acute periodontitis	**3.48 (2.37–5.11)**	**2.59 (1.91–3.51)**	**3.24 (1.22–8.57)**	**6.05 (3.01–12.17)**
Years of treatments				
Examinations (SA)	0.98 (0.86–1.11)	0.95 (0.86–1.04)	**0.78 (0.63–0.95)**	1.02 (0.85–1.22)
Preventive measures (SC)	1.06 (0.90–1.25)	**1.18 (1.05–1.32)**	0.92 (0.63–1.34)	0.83 (0.59–1.16)
Periodontal treatment (SD)	0.97 (0.86–1.09)	0.94 (0.86–1.03)	**1.17 (1.01–1.36)**	0.95 (0.82‐1.–10)
Restorative treatment (SF)	**0.86 (0.78–0.95)**	**0.90 (0.84–0.97)**	0.99 (0.86–1.14)	0.97 (0.85–1.11)
Tooth extractions (EBA)	**0.72 (0.60–0.87)**	**0.79 (0.68–0.93)**	1.10 (0.87–1.40)	1.15 (0.91–1.46)
Other diagnoses (ICD‐10)				
Type 1 or 2 diabetes mellitus (E10–E11)	0.94 (0.51–1.72)	0.55 (0.27–1.12)	1.10 (0.66–1.84)	0.78 (0.42–1.43)
Ischemic heart diseases (I20–I25)	1.19 (0.43–3.30)	1.69 (0.41–7.04)	1.01 (0.56–1.82)	0.99 (0.47–2.11)
Schizophrenia, schizotypal, delusional, and other non‐mood psychotic disorders (F20–F29 excluding F23)	1.20 (0.68–2.12)	1.17 (0.65–2.09)	0.71 (0.17–2.96)	0.82 (0.24–2.78)
Mood (affective) disorders (F30–F39)	**0.35 (0.13–0.95)**	0.86 (0.54–1.38)	0.72 (0.10–5.29)	2.01 (0.85–4.78)

*Note:* Statistically significant findings in bold.

## 4 Discussion

### 4.1 Key Findings

Among individuals who visited oral health care services in 2021–2022, the prevalence of periodontitis was quite low. Chronic periodontitis was diagnosed among less than 10%, while acute cases accounted for only a few percent. The number of years of having received different treatments during the follow‐up period (years 2014–2020) was associated with the odds of being diagnosed with periodontitis. For chronic periodontitis, preventive and restorative treatments were particularly associated with lower prevalence and incidence. In contrast, a history of periodontal treatments or tooth extractions, along with an earlier diagnosis, were associated with higher prevalence and incidence. For acute periodontitis, however, preventive care appeared to slightly increase the odds, while examinations and tooth extractions were associated with lower odds for the diagnosis. The results were similar among newly diagnosed patients, except in the cases of acute periodontitis diagnosis, where periodontal treatment was also associated with increased odds. These findings were in addition to other well‐established risk factors such as smoking and DM.

### 4.2 Strengths and Limitations

A major strength of the study is the comprehensive, population‐based data set, which includes the entire patient population within the public health care system. The use of a complete population‐based data set minimizes the risk of non‐response bias and enhances the real‐world relevance of the observed associations. We show results both from the fully adjusted and from age, sex, and their interaction adjusted analyses because when working with real‐world data from health care systems, extensive adjustment for a large number of factors may create a patient population that no longer reflects reality. Treatment need and care utilization are intrinsically related to patient characteristics, and if these characteristics are extensively controlled for, the interpretation and real‐world relevance of the observed associations become challenging.

Moreover, the data for this study were obtained from the Mediatri patient information system, which has proven exceptionally well‐suited for research purposes. This, for example, allowed us to account for non‐dental but established risk factors for periodontitis in the analyses, such as smoking and occurrence of other chronic conditions including diabetes, cardiovascular diseases, and severe mental disorders. Siun sote region's residents in general represent well urban, suburban, and rural Finnish populations. However, the demographic development of the Siun sote population has been characterized by an aging population, low birth rate, and a smaller proportion of individuals under 18 years compared with the total population. These factors may limit the generalizability of the findings.

In Finland, health care professionals are legally required to document each stage of patient care (the Client Data Act 2023). In oral health care, this responsibility lies with dentists, dental hygienists, and dental nurses. Documentation includes clinical findings, treatments, diagnoses (ICD‐10), and reasons for visits (ICPC‐2). Although these requirements have been in effect since 1995, the ICD‐10‐based prevalence was significantly lower than expected based on epidemiological studies and national clinical guidelines (Suominen et al. 2025; Working group set up by the Finnish Medical Society Duodecim and the Finnish Dental Society 2025). The number of new periodontitis diagnoses was relatively high at follow‐up. As there was likely no outbreak of chronic or acute periodontitis in North Karelia during years 2021–2022, this likely reflects changes in the utilization of diagnostic codes. This study included all oral health care visits in 2021–2022, regardless of the reason, and since only one primary diagnosis is required per visit, some conditions may have gone undocumented. Documentation challenges—such as information overload, inconsistent practices, and technical limitations—are common both nationally and internationally (Tokede et al. 2016).

In contrast, treatment codes are considered reliable due to national standardization and their role in salary calculations. Preventive care in this study included a wide range of actions, many of which are likely focused on caries prevention because patient guidance regarding periodontitis is included in periodontal treatment codes. This complicates the assessment of their impact in preventing periodontitis. In addition, preventive care codes are not used for salary payments, which likely makes the use of these codes more variable than others.

Adjusting for variables that may be mediators or colliders can bias regression coefficients and preclude causal interpretation (Westreich and Greenland 2013). Based on the DAG, we did not include any colliders or mediators in our regression analyses. However, a limitation is that we lack information on oral self‐care and socioeconomic factors. These factors were included only conceptually in the DAG, as no information on these factors was available in the register data used, being, however, a common pitfall in register‐based studies. Poor socioeconomic circumstances may limit access to oral health care services and the utilization of the services, as treatment costs remain a significant barrier in Finland. Although the entire population is entitled to publicly subsidized dental care, adult patients still bear a substantial share of costs, particularly in the private sector, where reimbursements cover only a small proportion of fees. Even in the public sector, increasing user charges may have limited the use of services for some individuals. However, the utilization of private oral health care services is strongly associated with higher socioeconomic status, particularly higher income and educational attainment, whereas public services are used relatively more frequently by individuals with lower incomes (Suominen et al. 2017).

We conducted separate, bivariate analyses for all participants and for those who had previously received oral health care within Siun sote. This approach was chosen to enable the assessment of the association between non‐attendance and periodontitis. It is a reality of public oral health care provision that only a proportion of the population uses oral health care services and some individuals receive care that is not captured in public health care records. It is a limitation that we lacked information on whether participants had sought care outside Siun sote during the follow‐up period from 2014 to 2020. Consequently, the potential contribution of private oral health care services could not be evaluated in this study. Nevertheless, this is unlikely to have introduced substantial bias, as previous evidence suggests that Finnish adults rarely switch dentists or receive oral health care concurrently from both the public and private sectors, also according to a recent report (Heinonen and Buri 2026). Furthermore, the findings were highly consistent between the analyses including all participants and those restricted to participants who had received treatments during 2014–2020.

### 4.3 Associations of Treatments With Periodontitis

A higher number of years receiving preventive or restorative care and undergoing examinations during the follow‐up period in 2014–2020 was associated with reduced odds of periodontitis diagnosis at the end of follow‐up in 2021–2022. This suggests that visiting oral health care services itself may be associated with a lower risk of periodontitis diagnosis, even though the initial periodontal health status of individuals remains unknown. It is possible that those who sought care were generally healthier than those who did not, and they may have remained healthier even without receiving any treatment. Interacting with dental professionals may provide an opportunity to maintain motivation and receive personalized guidance. This is supported by our finding that restorative treatment for caries was associated with lower occurrence of chronic and acute periodontitis. It is also possible that when restorative burden is low, focus shifts to periodontal health, which may increase the probability of diagnoses regardless of the actual periodontal status. In contrast, those who visit less often—often due to cost—are less likely to engage in preventive behaviors such as regular tooth brushing (Varela‐Centelles et al. 2020; Leggett et al. 2024).

Although preventive care—likely provided for reasons other than periodontal health—was offered significantly less frequently than other treatment types, it was associated with a lower risk of chronic periodontitis diagnosis, but not with acute periodontitis diagnosis. The finding that preventive care was associated with slightly higher odds of acute periodontitis may be attributable to the relatively small number of acute periodontitis cases and could therefore represent a chance finding. This interpretation is supported by the stratified analyses, in which the association was no longer statistically significant in any subgroup other than women under 65 years of age. Another explanation is that preventive care must also be appropriately targeted, and acute periodontitis often remains asymptomatic until it suddenly flares up, making early intervention more challenging. Individuals with more visible periodontal disease have likely received more attention, including preventive care. Current preventive codes (SC) are more commonly interpreted as targeting dental caries prevention, since the periodontal treatment codes (SD) also include patient guidance and maintenance therapy related to disease management. Earlier diagnoses and periodontal treatments reflect a disease history and highlight the chronic nature of periodontitis. Tooth extractions are associated with chronic periodontitis as a treatment for existing disease, whereas in the case of acute periodontitis, they may act as a preventive measure.

However, principles in the prevention of periodontal diseases include an appropriate periodontal diagnosis before submission to professional preventive measures and determining the selection of the type of preventive care. Also, preventive measures alone are not sufficient for treatment of periodontitis, and repeated and individualized oral hygiene instruction and professional mechanical plaque (and calculus) removal are considered important components of preventive programs (Tonetti et al. 2015). However, a review (Leggett et al. 2024) found that multiple factors influence whether prevention is delivered to patients, the most significant being environmental context and resources. This is seen in Finland, where the treatment provided by both the private and public sectors has historically focused on restorative care and does not meet the treatment needs identified in population studies. A recent dissertation (Linden 2023) examined the care received by patients in public oral health care between 2001 and 2013. For adults, the focus was still on filling teeth at the expense of periodontics, prosthetics, and prevention. Similar results have been reported in Sweden (Wänman and Wigren 1995), Norway (Fardal et al. 2020), and Ireland (Guiney et al. 2012), with significant discrepancies between the treatment needs identified in epidemiological studies and the care actually provided.

## 5 Conclusion

While the association of treatments was similar for prevalence and incidence, it differed between chronic and acute periodontitis diagnoses, probably highlighting their distinct pathophysiological profiles. Although preventive care is offered less frequently than other treatments, and likely often for reasons unrelated to periodontal health, it was associated with a lower risk of chronic but not acute periodontitis diagnosis.

## Author Contributions

S.A.L. contributed to conception, design, data acquisition and interpretation, and drafted and critically revised the manuscript. L.M.‐L. contributed to data acquisition and interpretation, performed all statistical analyses, and drafted and critically revised the manuscript. S.T., L.T., V.V., and P.J. contributed to conception, design, data acquisition and interpretation, and drafted and critically revised the manuscript.

## Ethics Statement

This study was conducted in full compliance with the Declaration of Helsinki (2008 version). As a register‐based study, it did not require ethical approval or informed consent under Finnish legislation. The study protocol was approved, and permission to use the register data was granted by the register administrator, Siun sote.

## Conflicts of Interest

The authors declare no conflicts of interest.

## Data Availability

The authors have nothing to report.
